# Exploring the heterogeneity of factors that may influence implementation of PrEP in family planning clinics: a latent profile analysis

**DOI:** 10.1186/s43058-021-00148-3

**Published:** 2021-05-04

**Authors:** Kaitlin N. Piper, Regine Haardörfer, Cam Escoffery, Anandi N. Sheth, Jessica Sales

**Affiliations:** 1 Department of Behavioral, Social, and Health Education Science, Rollins School of Public Health, Emory University, Atlanta, GA USA; 2 Department of Medicine, Division of Infectious Diseases, Emory University School of Medicine, Atlanta, GA USA

**Keywords:** Implementation science, PrEP, HIV/AIDS, Family planning, Consolidated Framework for Implementation Research, Latent profile analysis

## Abstract

**Background:**

Title X-funded family planning clinics have been identified as optimal sites for delivery of pre-exposure prophylaxis (PrEP) for HIV prevention. However, PrEP has not been widely integrated into family planning services, especially in the Southern US, and data suggest there may be significant implementation challenges in this setting. Because Title X clinics vary greatly in provider-, organizational-, and systems-level characteristics, there is likely variation in capacity to implement PrEP across clinics.

**Methods:**

We conducted a survey from February to June 2018 among providers and administrators of non-PrEP-providing Title X-funded clinics across 18 southern states. Survey items were designed using the Consolidated Framework for Implementation Research (CFIR) to assess constructs relevant to PrEP implementation. To explore the heterogeneity of CFIR-related implementation determinants and identify distinct sub-groups of Title X clinics, a latent profile analysis was conducted using nine CFIR constructs: complexity, relative advantage, cost, attitudes, implementation climate, compatibility, leadership engagement, available resources, and cosmopolitanism. We then conducted a multi-level analysis (accounting for nesting of participants within clinics) to test whether group membership was associated with readiness for implementation of PrEP, controlling for key sociodemographic characteristics.

**Results:**

Four hundred and fourteen healthcare providers/administrators from 227 non-PrEP-providing Title X clinics participated in the study. We identified six sub-groups of clinics that each had distinct patterns of PrEP implementation determinants. Clinic sub-groups included “Highest Capacity for Implementation”, “Favorable Conditions for Implementation”, “Mixed Implementation Context”, “Neutral Implementation Context”, “Incompatible Setting for Implementation”, and “Resource-Strained Setting”. Group membership was related to numerous provider-level (i.e., ability to prescribe medication) and clinic-level (i.e., provision of primary care) characteristics. In comparison to the “Neutral” group (which held neutral perceptions across the implementation determinants), the “Highest Capacity” and “Favorable Conditions” groups had significantly higher levels of implementation readiness, and the “Resource-Strained” group had a significantly lower level of implementation readiness.

**Conclusions:**

Latent profile analyses can help researchers understand how implementation readiness varies across healthcare settings, promoting tailoring of implementation strategies to unique contexts.

**Supplementary Information:**

The online version contains supplementary material available at 10.1186/s43058-021-00148-3.

Contributions to the literature

- Healthcare settings can exhibit significant heterogeneity in organizational context and barriers to implementation. Therefore, to promote intervention adoption, implementation plans and strategies may need to be tailored to each organization’s unique needs.
- Latent profile analyses can capture heterogeneity in organizational context across settings and promote the identification of unique sub-groups within the population.
- This study presents an example of how latent profile analysis can be used to identify sub-groups of clinics, allowing implementation planning to be tailored to the specific needs of unique segments of a diverse network of clinical sites.

## Background

Women account for approximately 20% of all new HIV infections in the United States (US), with a disproportionate number of new diagnoses occurring in the South [1, 2]. Effective HIV prevention efforts for women in the South are needed, not only to curb the epidemic among this population, but also to protect their sexual partners and prevent perinatal infection [1, 2]. HIV Pre-exposure prophylaxis (PrEP) is a safe and effective intervention to prevent HIV infection among women [3, 4]. However, PrEP utilization, access, and awareness remain low among women of all ages in the US [5–9]. For instance, in 2017, only 6% of PrEP users were women, and among the 176,670 heterosexual women for which PrEP was indicated, only 2% received a prescription [10–13].

Particularly for women in the South, a major barrier to PrEP utilization is low access to PrEP-providing clinics [13, 14]. PrEP is not widely offered in settings that women regularly receive sexual health and preventative services, such as women’s healthcare and family planning clinics [6, 13–15]. Integrating PrEP into settings where women regularly receive healthcare is a priority for mitigating the HIV epidemic among this population [16]. Specifically, Title X-funded family planning clinics in high HIV incidence settings (such as much of the Southern US) have been identified as optimal PrEP delivery sites for women. Title X family planning clinics are potentially ideal for expanding PrEP services for women, since they are an important safety net source of care and are already widely utilized by women for sexual health services, including HIV testing and education [17, 18]. There are approximately 4000 Title X-funded family planning clinics across the US, which serve over 4 million clients — the majority of which are low-income women [19, 20]. For many of these women, Title X clinics serve as their usual source of medical care, particularly in southern states that have not expand Medicaid [21, 22].

Integration of PrEP into new settings (such as family planning clinics) requires understanding of organizational context and potential obstacles to implementation [23]. To identify contextual factors related to implementation, theory-driven pre-implementation assessments, which employ surveys and interviews with clinical stakeholders, can provide critical insight into organizational context and capacity to implement evidence-based interventions (EBIs), such as PrEP. In prior work, we identified correlates of PrEP implementation capacity in Title X clinics, which included available resources (i.e., time, funding, and staffing), leadership engagement, implementation climate, and provider attitudes about PrEP [24]. Identifying barriers prior to adoption is critical, so that implementation plans, or “blueprints” for adoption of EBIs, can delineate strategies for overcoming known obstacles to implementation [25].

In addition to identifying key correlates of implementation capacity, implementation planning should also account for contextual variation between clinics [26–28]. Because healthcare settings (such as Title X clinics) can exhibit significant heterogeneity in organizational context and barriers to EBI implementation, “blueprints” may need to be tailored to each organization’s unique needs. For instance, clinical settings have shown wide variation in provider-level attitudes towards EBIs [29], organizational-level implementation climate [30], and systems-level policies and funding structures [31, 32]. Because of this variation, a “one-size-fits-all” approach to implementation planning may not support successful implementation of EBIs, such as PrEP.

In this study, we present a method for systematically capturing the contextual variation or heterogeneity in implementation determinants across healthcare organizations. Through the use of latent profile analysis (LPA) [33, 34], we identified sub-groups of Title X family planning clinics in the South, that each have unique profiles of implementation determinants (based on constructs from the Consolidated Framework for Implementation Research (CFIR) [35]). Identification of clinic sub-groups and their unique strengths/gaps allows researchers to better tailor implementation strategies and “blueprints” to the unique context of each group. Therefore, the objectives of this research are (1) identify sub-groups of Title X clinics based on their unique profiles of implementation determinants and (2) assess how sub-group membership is related to readiness for implementation of PrEP.

## Methods

### Study design and population

We invited healthcare providers and clinic administrators from Title X-funded family planning clinics in Department of Health and Human Services (DHHS) Regions III, IV, and VI to participate in an online survey from February through June 2018. These regions include states that comprise the Southern US including Alabama, Arkansas, Delaware, District of Columbia, Florida, Georgia, Kentucky, Louisiana, Maryland, Mississippi, New Mexico, North Carolina, Oklahoma, Pennsylvania, South Carolina, Tennessee, Texas, Virginia, and West Virginia. Providers were defined as individuals who have the potential ability to prescribe, counsel, or screen for PrEP. Clinic administrators were individuals who served in an administrative oversight capacity over the Title X activities in their clinic.

### Procedures

A comprehensive overview of the study’s protocol, methods, and measures has been published elsewhere [36]. In brief, quantitative surveys took place online with the assistance of the National Clinical Training Center for Family Planning (NCTCFP). The survey was emailed to the Title X clinic listserv for DHHS Regions III, IV, and VI, and listserv members received one to two email reminders per month. An advertisement for the survey was also posted on the NCTCFP website, and the survey was promoted though engagement with state Title X grant holders and through in-person recruitment at the biannual NCTCFP national meetings for Title X providers. The survey took approximately 15–25 min to complete, and participants received a $30 incentive. Institutional Review Boards from Emory University and University of North Carolina approved the study protocol prior to data collection (see Additional File 1 for STROBE checklist).

### Measures

A comprehensive overview of all measures as well as the full survey instrument are described elsewhere [36]. The survey assessed implementation determinants (from CFIR), readiness for PrEP implementation, and clinic and demographic characteristics. CFIR measures were selected based on a review of the PrEP implementation literature (to determine which implementation determinants are most relevant to PrEP) as well as discussions with Title X clinic providers, PrEP experts, and implementation scientists. Survey items were adapted from existing, validated measures of CFIR constructs [6, 37–39] or developed by our study team using CFIR-specific tools [35]. Prior to launching the survey, it was piloted with a small sample of Title X clinic providers and administrators. Pilot participants were interviewed about their experience taking the interview: their feedback was used to revise the survey and ensure questions were relevant and relatable to the focal population. We describe all of the measures below in detail. Table 1 also describes each construct and the associated survey items.

**Table 1:** Construct definitions and survey items

Construct/definition	Survey items
**Intervention characteristics**	
*Complexity*: Perceived difficulty of implementing PrEP services in their clinic^a^	1. Providing PrEP at my clinic seems easy to do^c^
*Relative advantage*: Perceived advantages of implementing PrEP in their clinic^b^	1. PrEP would be more effective than interventions we are currently promoting to prevent HIV among patients at our clinic
*Cost*: Perceived costs associated with implementing PrEP at their clinic^a^	1. PrEP is too expensive
**Characteristics of individuals**	
*Concerned attitudes*: Participant’s concerns about prescribing PrEP to patients in their clinic^a^	1. It is more suitable to provide PrEP in STD clinics than in family planning clinics 2. It is more suitable to provide PrEP in clinics that specialize in HIV care than in family planning clinics 3. Use of PrEP will increase HIV drug resistance 4. I am concerned that PrEP is not effective 5. Use of PrEP will result in less federal funding for HIV treatment 6. Non-biomedical (behavioral) HIV prevention interventions should be attempted before prescribing PrEP 7. Use of PrEP will cause patients to engage in riskier behaviors 8. If an HIV negative patient is in a relationship with an HIV positive partner, we should attempt treating partner instead of prescribing PrEP 9. If an HIV negative patient is in a relationship with an HIV positive partner, we should attempt treating partner before prescribing PrEP 10. I am concerned about potential side effects of PrEP
**Inner setting**	
*Implementation climate*: The extent to which PrEP implementation will be rewarded, supported, and expected within their clinic^b^	1. Individuals in my clinic will approve of providers prescribing PrEP to at-risk HIV-negative individuals 2. My clinic hires individuals who have previously used new types of HIV prevention practices 3. Individuals working in my clinic value new types of HIV prevention practices 4. Individuals working at my clinic are flexible enough to integrate new types of HIV prevention practices 5. Individuals working at my clinic are open to new types of HIV prevention practices
*Compatibility*: How PrEP aligns with the needs of the patient population served by the clinic^b^	1. PrEP seems like a good match for patients at my clinic 2. PrEP seems suitable for patients at my clinic. 3. Patients at my clinic who are at risk for HIV would really benefit from PrEP
*Leadership engagement*: How committed leadership is to improving clinic practices^b^	1. Senior leadership/clinical management in my clinic reward clinical innovation and creativity to improve patient care 2. Senior leadership/clinical management in my clinic solicit opinions of clinical staff regarding decisions about patient care 3. Senior leadership/clinical management in my clinic seek ways to improve patient education and increase patient participation in treatment
*Available resources*: The level of resources that could be dedicated to PrEP implementation at their clinic^b^	1. My clinic has necessary support in terms of budget or financial resources 2. My clinic has necessary support in terms of training 3. My clinic has necessary support in terms of facilities 4. My clinic has necessary support in terms of staffing
**Outer setting**	
*Cosmopolitanism*: The degree to which the clinic is networked with other external HIV or PrEP-providing organizations^b^	1. Individuals in my clinic are connected with other community organizations that provide HIV prevention services to patients
**Outcome**	
*Readiness for PrEP implementation*: Perceived clinic ability to conduct the various steps of the PrEP cascade^b^	1. Others in my clinic can screen a patient for symptoms of acute HIV 2. Others in my clinic can assess a patient’s HIV risk using the CDC PrEP guidelines 3. Others in my clinic can test a patient for HIV 4. My clinic has the capacity to provide HIV test results within one week of testing 5. Others in my clinic can assess a patients readiness for PrEP 6. Others in my clinic can assess a patients kidney function 7. My clinic has the capacity to conduct lab work to assess a patients kidney function and provide results within one week of testing 8. Others in my clinic can test a patient for active HBV infection and interpret results 9. My clinic has the capacity to provide HBV test results within one week of testing 10. Others in my clinic can ensure a patient is not taking any concomitant medications that may affect their ability to take PrEP 11. Others in my clinic can counsel a patient on the potential side effects of PrEP 12. Others in my clinic can counsel a patient on PrEP adherence 13. Others in my clinic can assess a patients pregnancy intentions and conduct preconception and contraceptive counseling 14. Others in my clinic can prescribe PrEP to a patient 15. Others in my clinic can help patients navigate insurance payments regarding PrEP treatment 16. Others in my clinic can refer patients to experts in PrEP and HIV when necessary 17. My clinic knows where to access resources for PrEP and HIV education 18. Others can conduct 3-month follow up visits for medication adherence counseling and side-effect assessment^d^ 19. Others can conduct 3-month follow up visits for laboratory testing^d^ 20. Others can conduct 3-month follow up visits for pregnancy intentions and preconception or contraceptive counseling^d^ 21. My clinic has an onsite pharmacy or affiliated pharmacy that will carry PrEP 22. There are community-based organizations or other partners in my community that will help facilitate PrEP access for patients at my clinic

^a^Higher values indicate more unfavorable scores (i.e., barriers)

^b^Higher values indicate more favorable scores (i.e., facilitators)

^c^Item was reverse-coded

^d^Administrators did not answer these questions

#### Implementation determinants

Implementation determinants selected for this study are based on constructs from CFIR [35], which provides a menu of 39 constructs that can be used as a practical guide for systematically assessing potential barriers and facilitators in preparation for implementing PrEP. Because it is often not practical or necessary to assess all constructs in a single study, evaluations typically focus on a subset of constructs [35]. Constructs for this analysis were selected based on their likelihood of (1) being a potential barrier (or facilitator) to PrEP implementation (as identified from the literature on PrEP implementation), and (2) having sufficient variation across clinics. For this analysis, nine different CFIR constructs were considered. The constructs covered four CFIR domains, including (1) intervention characteristics (i.e., complexity, relative advantage, and cost), individual characteristics (i.e., staff’s attitudes), inner setting factors (i.e., implementation climate, compatibility, leadership engagement, and available resources), and outer setting factors (i.e., cosmopolitanism) (Table 1). All CFIR-related survey items were evaluated on a 5-point Likert scale (1 = strongly disagree to 5 = strongly agree). Composite scores for each construct were calculated by taking the average of the contributing survey items. Most implementation determinants were adapted from validated measures (i.e., complexity, relative advantage, cost, implementation climate, leadership engagement, available resources, and PrEP attitudes) [6, 37–39]. Other measures that did not have validated scales (i.e., compatibility and cosmopolitanism) were developed by the study team based on CFIR definitions. Based on the results of our study, scales showed moderate to good internal consistency (Cronbach’s alpha: cost = 0.62, attitudes = 0.82, implementation climate = 0.83, compatibility = 0.88, leadership engagement = 0.90, available resources = 0.83).

#### Readiness for PrEP implementation

The primary outcome for this study is readiness for implementation of PrEP, which was measured based on steps of the PrEP delivery cascade (i.e., step 1, HIV risk assessment; step 2, PrEP education; step 3, PrEP eligibility assessment; step 4, PrEP prescription; and step 5, PrEP follow-up and monitoring) that the clinic could confidently implement. This measure is derived as a composite score based on the 22 or 19 (provider vs. administrator versions) survey items. The measure was developed by the study team since there were no previously-validated PrEP-specific measures of implementation readiness (survey items are depicted in Table 1). Responses to each of the survey items follow a Likert scale (1 = strongly disagree to 5 = strongly agree). Readiness for PrEP implementation was defined as the average score from the contributing survey items. Based on results of our study, the measure had high internal consistency (Cronbach’s alpha = 0.92).

#### Demographic and clinic/county characteristics

Individual and clinic-level characteristics were also assessed in the survey. Individual-level characteristics included self-reported age, race (white or non-white), ethnicity (Latinx or not Latinx), role (administrator or provider), years in role, and ability to prescribe medicine (yes or no). Clinic-level characteristics included metropolitan location (yes or no), pharmacy on-site (yes or no), staff to provide insurance support on-site (yes or no), and presence of primary care services (yes or no). Clinic metropolitan status was defined using the 2013 NCHS urban-rural classification scheme for counties, where metropolitan (urban) includes large central, fringe metro, medium metro, and small metro; and non-metropolitan (rural) includes micropolitan and noncore counties [40]. Additionally, clinic county characteristics included: HIV prevalence (per 100,000), percent of population under 200% of the federal poverty level, percent uninsured, percent White, percent Hispanic, and percent of women aged 15 to 44 (childbearing age) based on AIDSVu [41] and US Census data [42].

### Statistical analyses

There were 742 individuals who agreed to complete the survey. Of those, 519 (69.9%) respondents sufficiently completed the survey to warrant inclusion in our analyses. Our analyses used data from all surveys that met a minimal criteria for completeness (i.e., the respondent had to provide at least one response to questions related to PrEP use in their clinic). For the purposes of this pre-implementation analysis, the sample was restricted to clinics where PrEP was not currently being prescribed. Therefore, our sample included 414 participants from 227 non-PrEP prescribing Title X clinics. On average, there were 1.8 (range 1–12) participants per clinic. Clinic addresses were geocoded to identify participants residing in the same clinic, and each participant and clinic were given a unique identification number. Procedures for geocoding clinics have been described elsewhere [24].

Descriptive statistics were performed on all survey items (means (SD) or counts (%)). Pearson correlations were performed between all CFIR constructs to test for multicollinearity (see Additional File 2 for correlation matrix). Most CFIR measures were significantly correlated with each other; however, there were no strong correlations (all Pearson correlation coefficients are < |0.50|), so multicollinearity was not a major concern. The number of missing observations per variable ranged from 0 to 35. We used a non-parametric missing value imputation for mixed-type data (i.e., continuous and categorical) to impute missing values for all variables described above (including CFIR constructs, readiness for PrEP implementation, and demographics and clinic/county characteristics) (see “missForest” package for R [43]).

Using the nine CFIR constructs (complexity, relative advantage, cost, attitudes, implementation climate, compatibility, leadership engagement, available resources, and cosmopolitanism), LPA was performed in R using the “TidyLPA” package [44], to determine if Title X clinic providers and administrators coalesced into distinct sub-groups. LPA is a statistical method for dividing a population into mutually exclusive and exhaustive groups (or “latent profiles”). LPA identifies discrete groups of participants that share similar response patterns to a set of measures [33, 34]. Specifically, in this study, group membership is inferred based on participants’ response patterns to a set of CFIR constructs.

We investigated LPA solutions with one to ten groups. A one group solution would assume that all providers and administrators had similar perceptions across all CFIR measures, which was unlikely, and we assumed that solutions with more than 10 groups would have produced groups that were too small for generalization. To select the number of groups that fit the data best, we used four statistical metrics as well as interpretability of the groups. We considered solutions with high entropy levels (values > 0.80 indicate a high level of separation between the groups [45]), significant *p*-values for the bootstrap likelihood ratio test (BLRT) (which tests if the model performs significantly better than the *K*_−1_ group solution), and comparatively low AIC and BIC values. Simulation studies have shown that BIC and BLRT perform most reliably in latent profile analyses [46].

Based on the probabilities of class membership, each participant was assigned to a group they were most likely to belong. To understand the composition of each group, bivariate analyses were performed between group membership and individual/clinic-level characteristics (chi-squared was used for categorical variables and one-way ANOVA for continuous variables). We then conducted a random-intercept, multi-level model to test whether group membership predicted readiness for implementation of PrEP. This analysis modeled the nesting of participants within clinics. We fit the model to our data with SAS PROC GLMMIX using maximum likelihood estimation. The model controlled for variables that were significant in bivariate analyses (i.e., ability to prescribe medication, race, provision of primary care, racial/ethnic composition of county, county HIV prevalence, and percentage of women of childbearing age in the county).

## Results

### Participant characteristics

Of the 414 participants, the mean age was 45.92 (SD = 11.22) years. Participants were predominately White (*n* = 311, 75.12%) and most were clinic providers (*n* = 351, 84.78%). The mean number of years serving in their current role was 8.58 (SD = 7.89). Many clinics were located in metropolitan areas (*n* = 293, 70.77%) with high prevalence of HIV (mean = 459.78 cases/100,000, SD = 512.89 cases/100,000). See Table 2 for additional characteristics of the sample.

**Table 2:** Descriptive statistics, by group

	Mean (SD) or counts (%) by group							Full Sample
	Highest capacity (*n* = 17)	Favorable conditions (*n* = 105)	Mixed context (*n* = 53)	Neutral context (*n* = 201)	Incompatible setting (*n* = 26)	Resource-strained (*n* = 12)	*p*-value	*M* (SD)/*n* (%) (*n* = 414)
**CFIR constructs**								
Complexity^a^	1.71 (0.69)	2.67 (0.91)	3.83 (0.72)	3.18 (0.69)	4.27 (0.60)	4.08 (0.99)	< 0.001	3.17 (0.94)
Cost^a^	2.97 (0.58)	2.87 (0.44)	3.89 (0.43)	3.28 (0.38)	3.85 (0.56)	3.71 (0.57)	< 0.001	3.29 (0.55)
Concerned attitudes^a^	2.58 (0.71)	2.09 (0.45)	2.44 (0.51)	2.90 (0.44)	3.04 (0.42)	1.85 (0.47)	< 0.001	2.60 (0.60)
Advantage^b^	3.06 (1.20)	3.75 (0.79)	3.85 (0.74)	2.89 (0.76)	2.23 (0.76)	3.50 (1.24)	< 0.001	3.21 (0.94)
Climate^b^	4.04 (0.56)	3.74 (0.56)	3.58 (0.60)	3.18 (0.40)	2.62 (0.65)	2.30 (0.70)	< 0.001	3.35 (0.63)
Compatibility^b^	4.59 (0.52)	4.20 (0.43)	3.97 (0.45)	3.12 (0.46)	2.58 (0.63)	4.11 (0.73)	< 0.001	3.56 (0.74)
Leadership^b^	4.59 (0.48)	3.43 (0.77)	3.56 (0.82)	3.61 (0.70)	3.15 (0.88)	1.42 (0.59)	< 0.001	3.51 (0.85)
Resources^b^	4.32 (0.64)	3.04 (0.65)	2.42 (0.67)	2.99 (0.69)	2.11 (0.85)	1.58 (0.73)	< 0.001	2.89 (0.82)
Cosmopolitan^b^	4.65 (0.49)	3.77 (0.73)	4.26 (0.68)	3.77 (0.65)	2.85 (0.83)	1.75 (0.87)	< 0.001	3.75 (0.84)
**Outcome measure**								
Readiness	4.25 (0.39)	3.90 (0.49)	3.56 (0.71)	3.46 (0.54)	3.12 (0.64)	2.86 (0.45)	< 0.001	3.57 (0.62)
**Participant characteristics**								
Age	41.71 (13.13)	46.69 (11.09)	47.06 (13.27)	44.96 (10.54)	48.85 (9.43)	50.53 (12.84)	0.12	45.94 (11.22)
Role = administrator	2 (11.76)	7 (6.67)	10 (18.87)	38 (18.91)	4 (15.38)	2 (16.67)	0.12	63 (15.22)
Years in role	6.02 (6.17)	8.45 (7.51)	9.71 (7.52)	8.18 (8.26)	10.16 (7.65)	12.00 (8.22)	0.24	8.58 (7.89)
Ability to prescribe	6 (35.29)	52 (49.52)	33 (62.26)	51 (25.37)	14 (53.85)	10 (83.33)	< 0.001	166 (40.10)
Race = White	6 (35.29)	79 (75.24)	41 (77.36)	153 (76.12)	21 (80.77)	11 (91.67)	0.005	311 (75.12)
Ethnicity = Latinx	0 (0.0)	4 (3.81)	4 (7.55)	5 (2.49)	2 (7.69)	0 (0.0)	0.37	15 (3.62)
**Clinic characteristics**								
Pharmacy on-site	9 (52.94)	46 (43.81)	31 (58.49)	106 (52.74)	12 (46.15)	4 (33.33)	0.38	208 (50.24)
Insurance support on-site	16 (94.12)	87 (82.86)	40 (75.47)	160 (79.60)	19 (73.08)	8 (66.67)	0.37	330 (79.71)
Primary care provision	3 (17.65)	30 (28.57)	15 (28.30)	81 (40.30)	4 (15.38)	5 (41.67)	0.03	138 (33.33)
**County characteristics**								
Metropolitan	14 (82.35)	77 (73.33)	41 (77.36)	135 (67.16)	17 (65.38)	9 (75.0)	0.51	293 (70.77)
HIV prevalence	900.88 (889.05)	644.49 (625.34)	446.87 (464.22)	324.41 (386.33)	305.46 (156.34)	567.33 (462.73)	< 0.001	450.79 (512.89)
Percent poverty	20.43 (4.95)	18.45 (5.56)	18.07 (4.81)	18.57 (5.16)	18.51 (4.68)	18.17 (6.98)	0.74	18.53 (5.23)
Percent uninsured	11.48 (3.44)	12.25 (3.22)	13.17 (4.25)	13.22 (3.77)	13.24 (3.74)	12.39 (3.40)	0.15	12.87 (3.69)
Percent female (age 15–44)	21.67 (2.91)	21.12 (3.61)	20.66 (3.19)	19.43 (2.48)	20.07 (2.67)	19.93 (2.72)	< 0.001	20.17 (3.02)
Percent White	57.07 (23.10)	62.12 (21.89)	67.69 (16.53)	71.94 (17.39)	70.15 (13.66)	68.10 (22.07)	< 0.001	68.07 (19.19)
Percent Hispanic	6.98 (3.50)	8.03 (9.27)	11.46 (13.93)	8.15 (11.40)	15.35 (22.14)	6.59 (5.21)	0.03	8.90 (12.00)

^a^Higher values indicate more unfavorable scores (i.e., barriers)

^b^Higher values indicate more favorable scores (i.e., facilitators)

### Latent group membership

**Fig. 1:**
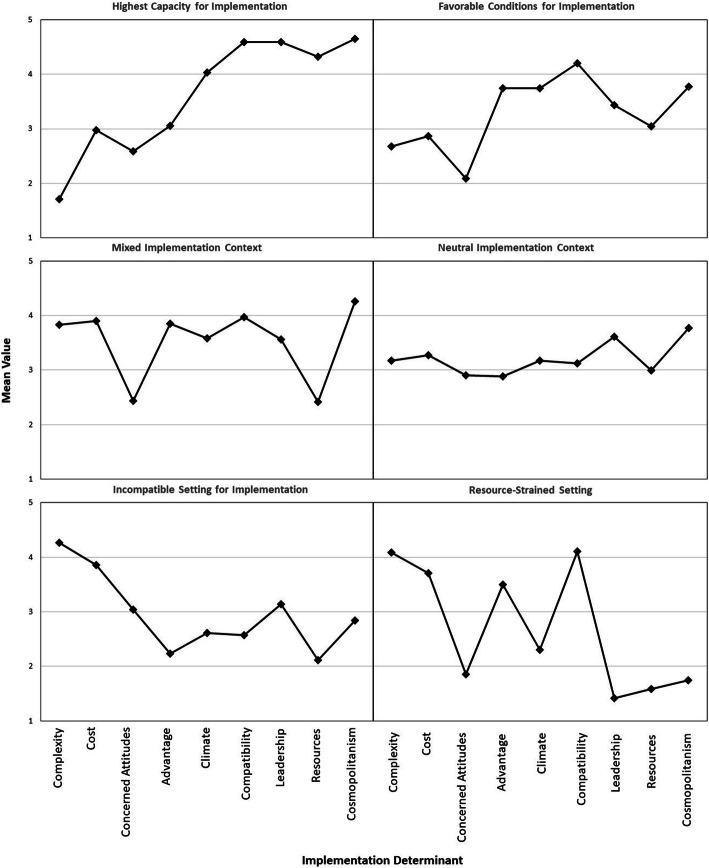
Profile plot: Mean scores across implementation determinants, by group. Results of the latent profile analysis indicate that 6 groups represent the heterogeneity in implementation determinants across Title X clinics in the South. For each group, the mean values for each implementation determinant are presented. Higher values for complexity, cost, and concerned attitudes indicate more unfavorable scores (e.g., barriers to implementation). Higher values for advantage, climate, compatibility, leadership, resources, and cosmopolitanism indicate more favorable scores (e.g., facilitators to implementation)

Based on entropy, BIC, AIC, BLRT, and interpretability, we selected the 6-group model (entropy = 0.81, AIC = 7880.3, BIC = 8154.1, BLRT *p*-value = 0.009) (see Additional File 3 for fit metrics for 1- to 10-group solutions). The 6-group model had high separation between groups (entropy > 0.80), the lowest BIC value, and significant BLRT *p*-value, indicating that the model performed significantly better than the 5-group solution. The 7-group solution had a lower AIC value compared to the six group model; however, we believed that the 7-group model was less interpretable and had small within-group sample sizes. We consulted with PrEP experts and interviewed Title X family planning providers and administrators to ensure that the 6-group solution was interpretable from a practice-based perspective. Below we describe each of the 6 different groups: (1) Highest Capacity for Implementation, (2) Favorable Conditions for Implementation, (3) Mixed Implementation Context, (4) Neutral Implementation Context, (5) Incompatible Setting for Implementation, and (6) Resource-Strained Setting (see Fig. 1 and Table 2).

#### Group 1: Highest capacity for implementation

Compared to all other groups, the High Capacity group (*n* = 17) had the highest scores across many implementation determinants, suggesting that this group has the best capacity for PrEP implementation (Fig. 1). Specifically, the inner and outer setting CFIR constructs all had mean scores above 4 (on a 5-point scale). Scores for inner setting factors indicate that these clinics highly value PrEP and HIV prevention (implementation climate mean = 4.04, SD = 0.56), believe that PrEP is compatible with their patient population (compatibility mean = 4.59, SD = 0.52), believe that their clinic leaders are highly supportive of adopting new practices (leadership engagement mean = 4.59, SD = 0.48), and have a high level of resources that could be dedicated to PrEP implementation (available resources mean = 4.32, SD = 0.64). Additionally, in the outer setting, they are highly networked with external HIV organizations (cosmopolitanism mean = 4.65, SD = 0.49). They also perceived PrEP implementation to have the lowest complexity (mean = 1.71, SD = 0.69) compared to other groups. Notably, this group included clinics that were most commonly from metropolitan areas, which had significantly higher HIV prevalence rates, higher levels of poverty, and higher proportion of minority residents compared to any other group. Nearly all of these clinics had insurance support on-site, and participants in this group tended to have less experience in their role and were more likely to be non-White (Table 2).

#### Group 2: Favorable conditions for implementation

This group (*n* = 105) had the second-most favorable profile compared to the High Capacity group (Fig. 1). Although the Favorable group scored relatively high, averages for the inner and outer setting constructs were nearly a full point lower than the High Capacity group (Table 2). Specifically, this group had the second-highest scores for implementation climate (mean = 3.74, SD = 0.56), compatibility (4.20, SD = 0.43), and available resources (3.04, SD = 0.65). In the intervention characteristics domain, this group had the lowest scores for cost (mean = 2.87, SD = 0.44), suggesting that they do not perceive the cost of PrEP to be a major barrier to implementation. They also believe PrEP would be easy to implement (second-lowest complexity score, mean = 2.67, SD = 0.91), and they are not concerned about providing PrEP to their clients (second-lowest concerned attitudes, mean = 2.09, SD = 0.45). Most of these clinics have insurance support on-site and are located in metropolitan areas with a high prevalence of HIV (Table 2).

#### Group 3: Mixed implementation context

Compared to the High Capacity and Favorable groups which had good scores across all implementation determinants, the Mixed Context group (*n* = 53) had both strengths and weaknesses (Fig. 1). Strengths included having the highest relative advantage score across all groups (mean = 3.85, SD = 0.74), suggesting that offering PrEP would be better than their current HIV prevention practices. This group also has strong connections to external HIV organizations (cosmopolitanism mean = 4.26, SD = 0.68), as well as appears to value HIV prevention (implementation climate mean = 3.58, SD = 0.60) and believes PrEP is well-suited for their patient population (compatibility mean = 3.97, SD = 0.45). However, the high complexity of offering PrEP (complexity mean = 3.83, SD = 0.72), high cost of PrEP (cost mean = 3.89, SD = 0.43), and lack of available resources for implementation (available resources mean = 2.42, SD = 0.67) could be barriers to adoption in this group. Many of the individuals in this group have the ability to prescribe medication, and this group has the highest percentage of clinics that have a pharmacy on-site.

#### Group 4: Neutral implementation context

This group (*n* = 201) had very average scores across all of the CFIR-related determinants of implementation (means ranged from 2.9 to 3.77) (Fig. 1). Compared to the other groups, it does not have strong strengths or strong weaknesses. Though, it has the second-highest score for leadership engagement (mean = 3.61, SD = 0.70), and clinic administrators most commonly fell in this group. Notably, clinics that provided primary care were most likely to be in this group, and most of these clinics had insurance support on-site. Compared to the aforementioned groups, the Neutral group had a higher representation of non-metropolitan communities, which had lower HIV prevalence rates and more White residents (Table 2).

#### Group 5: Incompatible setting for PrEP implementation

The Incompatible group (*n* = 26) had the lowest scores for many of the implementation determinants, especially in the Intervention Characteristics and Characteristics of Individuals domains (Fig. 1). Compared to all other groups, they had the most negative attitudes about PrEP (concerned attitudes mean = 3.04, SD = 0.42), and they were concerned that PrEP would be too hard to offer in their clinic (complexity mean = 4.27, SD = 0.60) and would be too costly (cost mean = 3.85, SD = 0.56). This group was also most concerned that PrEP did not align with the needs of their clients (compatibility mean = 2.58, SD = 0.63), and they did not think it had an advantage for their patients (relative advantage mean = 2.23, SD = 0.76). This group had the highest representation of clinics from non-metropolitan areas, which had the lowest HIV prevalence rates (Table 2).

#### Group 6: Resource-strained setting

Lastly, the Resource-Strained group (*n* = 12) noted major obstacles in the Inner Setting and Outer Settings domains (Fig. 1). Specifically, this group had the lowest resources for implementation (available resources mean = 1.58, SD = 0.73), most unsupportive leadership (leadership engagement mean = 1.42, SD = 0.59), worst climate for implementation (mean = 2.30, SD = 0.70), and lack of partnerships with external HIV organizations (cosmopolitanism mean = 1.75, SD = 0.87). Because of these major barriers, they also thought PrEP would be too complex to implement in their clinic (complexity mean = 4.08, SD = 0.99). However, in striking opposition to the Incompatible group, the Resource-Strained group had least concerned attitudes about PrEP (mean = 1.85, SD = 0.47), compared to any other group. They also believe PrEP would be highly advantageous (relative advantage mean = 3.50, SD = 1.24) and would be a great benefit to their clients (compatibility mean = 4.11, SD = 0.73). The individuals in this group had the most years of experience, and the majority of these participants were able to prescribe medication. Clinics in this group were commonly from metropolitan areas with a high prevalence of HIV (Table 2).

### Readiness for implementation and group membership

**Fig. 2:**
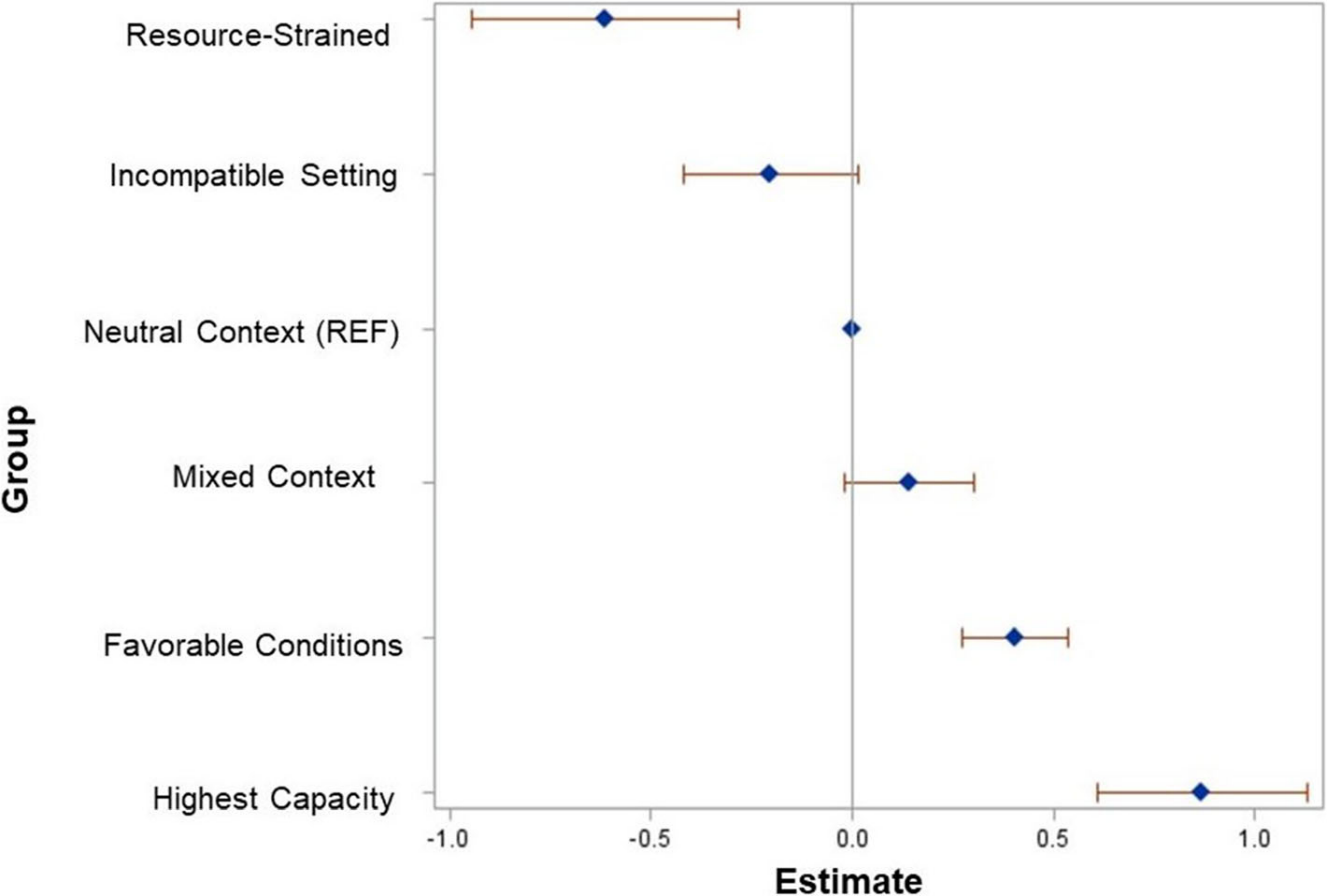
Results of generalized linear mixed model, assessing the relationship between group membership and readiness for implementation, controlling for covariates. Diamonds represent unstandardized *β*’s and bars represent 95% CI

Compared to the Neutral group, the Highest Capacity (*B* = 0.86, SE = 0.13, *p* < 0.0001) and Favorable (*B* = 0.40, SE = 0.07, *p* < 0.0001) groups had significantly higher levels of implementation readiness; and the Resource-Strained group (*B* = − 0.61, SE = 0.17, *p* = 0.0003) had significantly lower levels of implementation readiness (see Fig. 2, full results of the multi-level model are presented in Additional File 4). The ICC was 0.34, indicating that 34% of the variation in readiness for implementation of PrEP was at the clinic-level.

## Discussion

LPA is a useful method for capturing the variation in implementation context and barriers to adoption of new practices across sites. In this study, we identified six different sub-groups of Title X-funded family planning providers and administrators in the Southern US that have unique profiles of barriers and facilitators to PrEP implementation. Two groups (“Highest Capacity” and “Favorable Conditions”) showed significantly increased levels of readiness for implementation while one group (“Resource-Strained”) showed significantly reduced levels of readiness for implementation of PrEP. To enhance the likelihood of PrEP implementation success in each of these groups, we can employ tailored implementation strategies for overcoming identified weaknesses and leveraging strengths [26, 47, 48].

Both the “Highest Capacity” and “Favorable” groups (encompassing about 30% of the sample (*n* = 122)) have high scores across implementation determinants related to the intervention (i.e., PrEP), the clinic, the individuals working within the clinic, and the clinic’s external network. These clinics are also commonly located in metropolitan areas with the highest HIV prevalence rates, suggesting that they have an opportunity to reach a particularly at-risk population. These may be ideal clinics to begin the roll-out of PrEP services, since their context is likely able to support PrEP implementation. However, we suggest that all clinics, prior to PrEP implementation, receive a training on the process for conducting the PrEP cascade (i.e., HIV risk assessment, PrEP patient education, assessing patient eligibility for PrEP, PrEP prescription, and PrEP follow-up care) to ensure that clinic staff, providers, and administrators are informed of necessary requirements. We have previously shown that a 1 hour training is able to significantly increase providers’ PrEP knowledge, attitudes, and skills [49].

However, the remainder of the groups may need additional implementation strategies beyond training. For instance, the “Mixed Context” group (*n* = 53, 12.8%), although they believe PrEP offers advantages over their current HIV prevention services, perceives the cost of PrEP to be a major obstacle to implementation. This is understandable as the South has a high proportion of uninsured patients and many states have not expanded Medicaid [50], which can hinder the affordability of PrEP for low-income and vulnerable patients. To overcome cost hurdles, there are some services in place, such as HHS’s “Ready, Set, PrEP” Program and Gilead’s Advancing Access Program, which cover the cost of PrEP prescriptions for uninsured patients. Gilead also has a co-pay program to help insured patients cover the cost of co-pays and deductibles for PrEP services. Many of the “Mixed Context” clinics already have staff on site that assist patients with insurance enrollment (75%), and these insurance navigators should receive education on procedures for enrolling patients in these PrEP payment assistance programs. Additionally, clinics that are concerned about the cost of PrEP and have limited financial resources can also consider pursuing grant funding for PrEP services, as this has been a facilitator for implementation in other family planning clinics [15, 51].

Furthermore, for successful PrEP implementation to occur in the “Incompatible Setting” and “Resource-Strained” groups, which represent about 9% of the sample (*n* = 38), significant amounts of system strengthening as well as building a “business case” for PrEP delivery are likely needed. Specifically, the “Incompatible Setting” group had the most negative attitudes about PrEP and perceived that PrEP was not compatible with the needs of their patient population. Clinics in this group were more likely to be located in non-metropolitan communities with the lowest rates if HIV (compared to other groups), so HIV prevention may be less of a priority for these providers. However, these negative attitudes about PrEP are concerning, given that the Southern US is the current epicenter of the HIV epidemic [2]. Providers and staff in this group may require additional education on the prevalence and impacts of HIV to overcome knowledge/attitude barriers. In addition, the “Incompatible Setting” group believed that PrEP implementation was too complex. Although there is limited guidance on strategies to combat issues related to complexity, expert recommendations suggest that “promoting adaptability” may work for these settings [47]. Promoting adaptability requires tailoring the intervention (i.e., PrEP) to the site’s needs [25]. For instance, our research in family planning clinics has shown that some clinics have decided to provide the initial steps of the PrEP cascade (HIV risk assessment and PrEP patient education) and then refer patients to external providers for PrEP prescriptions and continued care [15, 51]. Clinics in the “Incompatible Setting” group may consider this referral adaption, so that women who could benefit from PrEP are at a minimum educated and made aware of the availability of PrEP services in their community. Universal education pertaining to PrEP is especially important given consistently low knowledge and awareness about PrEP reported among women in the South [5, 7]. Other potential adaptations to PrEP, that could help overcome barriers, include pharmacy-based PrEP services (which could be useful for clinics that have a pharmacy on-site) [52], telemedicine [53], and at-home PrEP services [54].

However, in the “Resource-Strained” group it may be unlikely that any PrEP services could be offered without significant additional internal and external systems changes. This group has clinic characteristics (low implementation climate, available resources, leadership engagement) that are very unfavorable for successful implementation. They also have very low cosmopolitanism scores, suggesting that even the adaptation strategy would be ineffective, since they do not have relationships with external HIV organizations where they could refer patients. Not only would these clinics need to build partnerships with external HIV organizations and PrEP providers, they would also need to identify strong champions to lead the adoption of PrEP services in their clinic (to overcome climate and leadership weaknesses) and pursue grant funding (to overcome cost and resource barriers) [47].

Future research should focus on the provision of these tailored implementation plans to each sub-group of clinics. Evaluations would yield important data on the success of these implementation strategies to promote PrEP adoption and sustained delivery in each clinic sub-group. Limitations of this study include the use of a non-probability sampling method and self-reported data (i.e., not based on observations of clinical context). Additionally, some of the groups had small within-group sample sizes, so these groups may not generalize to the larger population of Title X clinics. Furthermore, we were unable to examine all CFIR constructs. CFIR is comprised of 39 different constructs and although our survey captured many of these, we were unable to utilize all constructs in the LPA. If too many constructs are included in this analysis, it can become difficult to interpret; therefore, we selected nine CFIR constructs that we hypothesized to have the greatest impact on PrEP implementation as well as the greatest variability across clinical settings. These decisions were made based on a review of the PrEP implementation literature and discussions with experts in implementation science and HIV prevention as well as clinical providers. Despite limitations, the LPA approach may be especially helpful for large public health systems, like the Title X Family Planning Program, which provides funding for the provision of family planning and other preventative health services for un-/under-insured individuals through a network of approximately 4000 clinical sites across the US. Learning if there are systematic differences within the network that could support more tailored implementation planning might allow for these networks to more easily scale interventions across sites, especially when interventions may be required at the Title X level.

## Conclusion

In conclusion, we identified six different groups of Title X family planning clinics, based on their profiles of determinants of PrEP implementation. Some groups had very favorable scores across the implementation determinants as well as high readiness for implementation, suggesting an ideal environment for roll-out of PrEP services. Other groups, with identified contextual weaknesses, would likely need system strengthening prior to PrEP implementation along with tailored implementation strategies to overcome barriers to successful PrEP implementation. LPA has important implications for engaging with complexity in implementation research and understanding the contextual variation across sites. This method also promotes identification of sub-groups, allowing implementation planning to be tailored to the specific needs of unique segments of this diverse network of clinical sites comprising the Title X family planning safety net system.

## Supplementary Information

**Additional file 1:.** STROBE Statement—Checklist of items that should be included in reports of cross-sectional studies

**Additional file 2: **Pearson correlations between CFIR constructs (*n*=414)

**Additional file 3:.** Model fit metrics

**Additional file 4:.** Results of multi-level model predicting readiness for implementation of PrEP

## Data Availability

The datasets analyzed during the current study are available from the corresponding author on reasonable request.

## Abbreviations

AIC: Akaike information criterion
BIC: Bayesian information criterion
BLRT: Bootstrap likelihood ratio test
CFIR: Consolidated framework for implementation research
EBIs: Evidence-based interventions
HIV: Human immunodeficiency virus
LPA: Latent profile analysis
NCTCFP: National clinical training center for family planning
PrEP: Pre-exposure prophylaxis
US: United States
USDHHS: United States department of health and human services
