# Supplementary material for: Exploring the heterogeneity of factors that may influence implementation of PrEP in family planning clinics: a latent profile analysis
Source: Implement Sci Commun. 2021 May 4;2:48. doi: 10.1186/s43058-021-00148-3 (PMC8097793; doi:10.1186/s43058-021-00148-3)
Supplement: Supplementary file 2 — Additional file 2: Pearson correlations between CFIR constructs (n=414) [file 43058_2021_148_MOESM2_ESM.docx]

|  | **Complexity** | **Advantage** | **Cost** | **Attitudes** | **Climate** | **Compatibility** | **Leadership** | **Resources** | **Cosmopolitan** | **Readiness** |
| --- | --- | --- | --- | --- | --- | --- | --- | --- | --- | --- |
| **Complexity** | 1 |  |  |  |  |  |  |  |  |  |
| **Advantage** | -0.13** | 1 |  |  |  |  |  |  |  |  |
| **Cost** | 0.39*** | -0.03 | 1 |  |  |  |  |  |  |  |
| **Attitudes** | 0.09 | -0.22*** | 0.25*** | 1 |  |  |  |  |  |  |
| **Climate** | -0.24*** | 0.15** | -0.21*** | -0.19*** | 1 |  |  |  |  |  |
| **Compatibility** | -0.28*** | 0.43*** | -0.20*** | -0.44*** | 0.43*** | 1 |  |  |  |  |
| **Leadership** | -0.12* | 0.01 | -0.06 | 0.11* | 0.29*** | -0.05 | 1 |  |  |  |
| **Resources** | -0.29*** | 0.02 | -0.31*** | 0.03 | 0.20*** | 0.03 | 0.48*** | 1 |  |  |
| **Cosmopolitan** | -0.10* | 0.04 | 0.01 | -0.08 | 0.31*** | 0.15** | 0.35*** | 0.14** | 1 |  |
| **Readiness** | -0.27*** | 0.18*** | -0.26*** | -0.24*** | 0.36*** | 0.35*** | 0.31*** | 0.36*** | 0.26*** | 1 |

Additional File 2. Pearson correlations between CFIR constructs (n=414)

Note: *<0.05, **<0.01, ***<0.001
