# Supplementary material for: Exploring the heterogeneity of factors that may influence implementation of PrEP in family planning clinics: a latent profile analysis
Source: Implement Sci Commun. 2021 May 4;2:48. doi: 10.1186/s43058-021-00148-3 (PMC8097793; doi:10.1186/s43058-021-00148-3)
Supplement: Supplementary file 3 — Additional file 3:. Model fit metrics [file 43058_2021_148_MOESM3_ESM.docx]

Additional File 3: Model fit metrics

| **Number of Groups** | **AIC** | **BIC** | **Entropy** | **BLRT**  **p-value** |
| --- | --- | --- | --- | --- |
| 1 | 8512.33 | 8584.8 | 1 | NA |
| 2 | 8236.42 | 8349.14 | 0.73 | 0.009 |
| 3 | 8044.14 | 8197.13 | 0.84 | 0.009 |
| 4 | 7982.67 | 8175.91 | 0.78 | 0.009 |
| 5 | 7941.58 | 8175.08 | 0.79 | 0.009 |
| 6 | 7880.34 | 8154.1 | 0.81 | 0.009 |
| 7 | 7843.11 | 8157.13 | 0.83 | 0.009 |
| 8 | 7847.64 | 8201.92 | 0.77 | 0.32 |
| 9 | 7801.77 | 8196.30 | 0.78 | 0.009 |
| 10 | 7808.12 | 8242.92 | 0.76 | 0.61 |
