# Supplementary material for: Exploring the heterogeneity of factors that may influence implementation of PrEP in family planning clinics: a latent profile analysis
Source: Implement Sci Commun. 2021 May 4;2:48. doi: 10.1186/s43058-021-00148-3 (PMC8097793; doi:10.1186/s43058-021-00148-3)
Supplement: Supplementary file 4 — Additional file 4:. Results of multi-level model predicting readiness for implementation of PrEP [file 43058_2021_148_MOESM4_ESM.docx]

Additional File 4. Results of multi-level model predicting readiness for implementation of PrEP

|  | **Model 1** (Unconditional Model) | **Model 2** (group as only predictor) | **Model 3** (controlling for individual level factors) | **Model 4** (controlling for individual and clinic level factors, final model) |
| --- | --- | --- | --- | --- |
| **Fixed Effects** |  |  |  |  |
| Individual Level Variables |  |  |  |  |
| Intercept | 3.58*** | 3.46*** | 3.52*** | 3.30*** |
| Group |  |  |  |  |
| Highest Capacity |  | 0.89*** | 0.86*** | 0.86*** |
| Favorable Conditions |  | 0.40*** | 0.39*** | 0.40*** |
| Mixed Context |  | 0.13 | 0.11 | 0.14 |
| Neutral Context |  | Ref | Ref | Ref |
| Incompatible Setting |  | -0.26* | -0.27* | -0.20 |
| Resource-Strained |  | -0.56** | -0.58*** | -0.61*** |
| Race=White |  |  | -0.11 | -0.08 |
| Ability to Prescribe Medication=Yes |  |  | 0.07 | 0.06 |
| Clinic Level Variables |  |  |  |  |
| Primary Care Provision= Yes |  |  |  | 0.27*** |
| HIV County Prevalence |  |  |  | 0.00006 |
| Percent Female (age 15-44) in County |  |  |  | 0.002 |
| Percent White in County |  |  |  | 0.001 |
| Percent Hispanic |  |  |  | -0.01** |
| **Random Effects** |  |  |  |  |
| Level 1 (between individual) variance | 0.25* | 0.20* | 0.20* | 0.20* |
| Level 2 (between clinic) variance | 0.13* | 0.12* | 0.11* | 0.09* |
| **Model Fit** |  |  |  |  |
| Deviance | 747.76 | 663.96 | 667.63 | 693.48 |

Note: All models account for clustering of participants. p<0.05* p<0.01** p<0.001***
